# Metagenomic profiling for assessing microbial diversity and microbial adaptation to degradation of hydrocarbons in two South African petroleum-contaminated water aquifers

**DOI:** 10.1038/s41598-018-25961-0

**Published:** 2018-05-15

**Authors:** Leonard Kachienga, Keshri Jitendra, Maggy Momba

**Affiliations:** 1 Department of Environmental, Water and Earth Sciences, Faculty of Science, Tshwane University of Technology, Arcadia Campus, P/Bag X680, Pretoria, 0001 South Africa; 2 Institute of Postharvest and Food Sciences, Agricultural Research Organization, Volcani Centre, 50250 Bet Dagan, Israel

## Abstract

Biodegradation of hydrocarbons by indigenous populations of microorganisms found in petroleum-contaminated water sources represents one of the primary mechanisms by which petroleum and other hydrocarbon pollutants are eliminated from the aquatic environment. The identification of these microorganisms, which have capabilities to convert the majority of toxic hydrocarbons into compounds that are less harmful for end-users, is therefore crucial for bioremediation purposes. The aim of this study was to profile the microbial diversity of two South African petroleum-contaminated water aquifer sites and to determine the microbial adaptation to hydrocarbon degradation using a metagenomics approach. The sequenced samples revealed that protozoa (62.04%) were found to be the most dominant group, followed by fungi (24.49%), unknown (12.87%), and finally other sequences such as Animalia and plantae which were <(0.10%) domains in the first oil-polluted aquifer site. In the second site, protozoa (61.90%), unknown (16.51%), fungi (11.41%) in that order. According to the classification at the genus level, the dominant group was *Naegleria* (15.21%), followed by *Vorticella* (6.67%) as the only ciliated protozoan genus, other species such as *Arabidopsis* (2.97%), *Asarum* (1.84%) *Populus* (1.04%) were significantly low and drastically lower in the first site. Regarding the second site, the dominant group was *Naegleria* (18.29%) followed by *Colpoda* (9.86%) with the remainder of the genera representing <2%. Overall results demonstrated the ability of various groups of microorganisms to adapt and survive in petroleum oil-polluted water sites regardless of their respective distributions and this can be explored further for their role in bioremediation and environmental management.

## Introduction

Continuous pollution of the existing freshwater sites across the globe by hydrocarbons of petroleum and their by-products has disastrous consequences for the end-users such as animals, humans, plants and microorganisms. According to Haritash and Kaushik^1^, the ongoing pollution by these hydrocarbons and their derivatives results in the accumulation of xenobiotics and other toxic compounds in both water sources and the entire environmental landscape. Petroleum is a complex mixture of hydrocarbons and non-hydrocarbons from metallic porphyrins, acid and organometallic compounds origin^2^. Polycyclic aromatic hydrocarbons (PAHs) are among the toxic components of petroleum oil spills considered to be the main contributor of mutagenicity and teratogenicity in humans^1^. Widespread oil spill pollution in various water sources has been reported during the 20^th^ century; oil pollution has been found to be mainly of anthropogenic origin, such as leaks and spills due to oil refining, handling, storage and transport from the refineries to the point of usage^3^. Millions of tons of petroleum oil are being exploited as a source of energy for the ever-increasing energy demand by the rapidly growing world population^4^. Microbial diversity involved in bioremediation is always considered as a form of natural degradation of hydrocarbons in various water sources^3^. The use of bioremediation techniques, which are mainly based on the use of microorganisms to biodegrade the crude oil contaminants, has shown signs of being a promising technology due to its overall high efficiency and cost-effectiveness^5^. Several microorganisms have diverse metabolic capabilities to degrade and utilise petroleum hydrocarbons as their carbon and energy sources via aerobic or anaerobic pathways^6–8^.

The focus of the biodegradation of aliphatic and aromatic hydrocarbons has been on using a special community of microorganisms, which have the genes of interest^9^. To date, only scant information is available regarding the interaction of microbial functions and their metabolic diversity during biodegradation by various microbial populations in any affected environment^10^. The introduction of metagenomics can play a vital role in unearthing and monitoring the microbial communities by providing access to the taxonomic and functional gene composition^10^. According to Gong *et al*.^11^, most of the metagenomic analysis tools have opened new windows of opportunities for researchers to analyse the microbial community as a whole (whole-genome sequencing) and the genetic diversity, which facilitates active metabolic pathways in any given environment.

While studies in the past have focused on species composition of a community, metagenomic studies (a gene-centric approach) enable assessment of the biological function of the gene rather than the taxonomic identity. The study of microbial biodiversity and whole-genome sequencing (WGS) and analysis of any complicated samples with numerous microorganisms, which are not pure and unculturable in any given laboratory, have made shotgun metagenomics a more efficient tool than most of the already existing conventional techniques^4^. Furthermore, there is always a well-established direct correlation between microbial diversity and the presence of hydrocarbons in any oil-contaminated sites. These studies have reported microbial species richness and diversity in different geographical locations. Furthermore, large variations have been observed in the functional capacity of the microbial communities identified at each location. Metagenomic studies therefore provide excellent opportunities for finding new microbial strains and genes involved in bioremediation of hydrocarbon contaminants. Therefore, the aim of this study was to assess the taxonomic profile of the metagenome and the microbial adaptations in two different petroleum-contaminated sites using a metagenomics approach.

## Materials and Methods

### Sample collection

The study was carried out between June and July 2016 and the petroleum-contaminated water samples were collected from two different petroleum-polluted water aquifers in Gezina (S -25.7°17′28.8, E 28.2°05′75.8), a suburb of Pretoria, South Africa. This is the only area in Pretoria where high-molecular-weight hydrocarbon oil spills or leakages from petroleum stations are discharged into water aquifers. Contaminated water samples, which were collected in 1 L sterile plastic bottles, were transported from the affected sites to the Tshwane University of Technology laboratory under standard refrigerated (−20 °C) conditions. To ensure the quality of collected water samples, the petroleum-contaminated water samples were thoroughly mixed prior to use.

### DNA extraction

The samples were filtered using filter paper with a pore size of 0.45 µM (Whatman Filter Paper No. 1). Two hundred microlitres (200 µL) of PBS together with 750 µL of lysis solution were added to the residue and thoroughly mixed. The metagenomic DNA was extracted independently for all the samples with ZR fungal/bacterial DNA Kit (Zymo Research), as per manufacturer’s instruction. Finally, the DNA was eluted in 30 µL of ultrapure water (Milli-Q water). The integration of the metagenomic DNA was assessed on the 0.8% agarose gel. The DNA quantity, quality and purity were measured using a NanoDrop ND-1000 spectrophotometer (PeqLab, Germany) where a 260/280 ratio of ~1.8 was accepted as “pure” for DNA.

### PCR amplification

The PCR amplification was performed on the extracted DNA samples using a combination of the universal 18 S rRNA gene forward primer 5′-GACGGGCGGTGTGTACA-3′ and the reverse primers 5′-CTGGTTGATCCTGCCAG-3′ or 5′-TGATCCTTCYGCAGGTTCAC-3′, respectively^12^. These primers were used to amplify approximately 500 bp of the 18 S rRNA gene sequence. For the final reaction, each PCR reaction mixture (50 µL) contained DreamTaq^TM^ Green DNA polymerase (dNTPs and 4 mM MgCl_2_), Master Mix (2×) (25 µL), nuclease-free water (22 µL), forward primer (1 µL) (0.2 µM) and reverse primer (1 µL) (0.2 µM) and metagenomic DNA (50–100 ng). The PCR cycling parameters used were as follows: initial denaturation step at 95 °C for 5 min, followed by 30 cycles of 94 °C for 1 min, 55 °C for 30 s and 72 °C for 1 min 30 s, with a 10 min extension at 72 °C, that was finally followed by rapid cooling to 4 °C. The PCR products were loaded into a 1% agarose gel and then visualised with an ultraviolet (UV) transilluminator (InGenius Bio Imaging System, Syngene, Cambridge, UK). The DNA was finally re-amplified with same sets of the two primers. The pooled samples were sequenced on the GS-FLX-Titanium series 454/Roche by Inqaba Biotechnology, South Africa.

### Sequencing of genomic DNA

Genomic DNA was sent to Inqaba Biotechnical Industries, a commercial NGS service provider, for sequencing. Briefly, genomic DNA samples were PCR amplified using a universal primer pair (566 F and 1200 R - targeting the 18 S rRNA gene). Resulting amplicons were gel purified, end repaired and illumina specific adapter sequence were ligated to each amplicon. Following quantification, the samples were individually indexed, and another bead based purification step was performed. Amplicons were then sequenced on illumina’s MiSeq platform, using a MiSeq v3 (600 cycle) kit. 20 Mb of data (2 × 300 bp long paired end reads) were produced for each sample. The BLAST-based data analysis was performed using an Inqaba in-house developed data analysis pipeline.

### Statistical analysis

The data were statistically analysed using the Stata computer software (version: STATA V12, STATA Corp. LP, 2012). Analysis of variance (ANOVA) was performed to compare the average percentage abundance between different microbes in two petroleum spill sites.

## Results

### Domain-level microbial communities in petroleum oil-contaminated water aquifers

**Figure 1:**
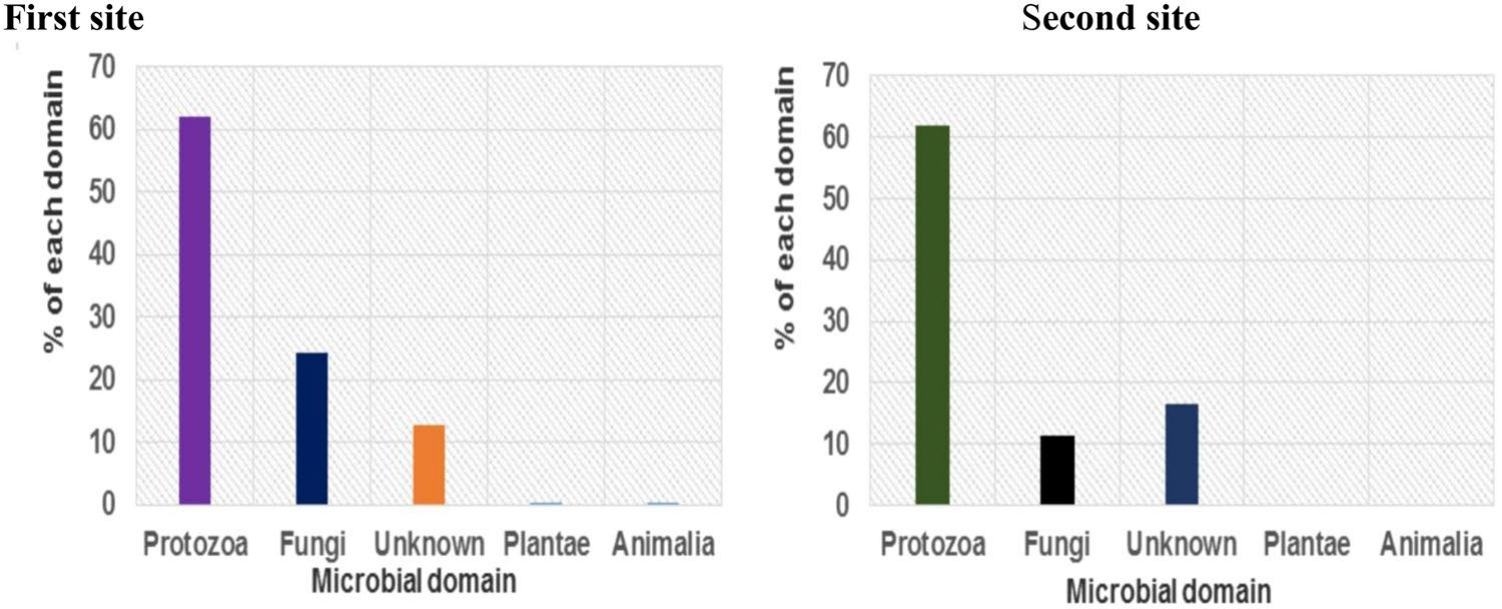
The different microbial domains observed in the first and second sites in petroleum-polluted water aquifers.

The various domains of microbes from two different petroleum oil-contaminated water sites are clearly illustrated in Fig. 1. In the first site, protozoa (62.04%) were found to be the most dominant group, followed by fungi (24.49%), unknown (12.87%) and finally other sequences such as Animalia and plantae which were below (0.10%). In the second site, the microbial communities were identified in the following order: protozoa (61.90%), unknown (16.51%) and fungi (11.41%) domains (Fig. 1). Statistically, there was no significant difference at *p* < 0.05 in terms of the domains of microbes observed in these two sites.

### Phylum-level microbial diversity of petroleum oil-polluted water sites

**Figure 2:**
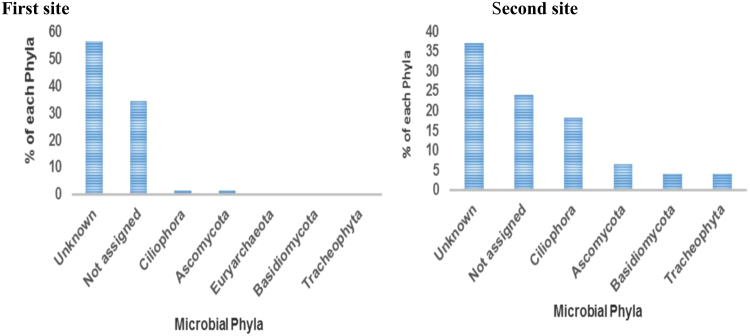
The different microbial phyla observed in the first and second site in petroleum-polluted water aquifers.

A representation of phylum-level microbial diversity identified in two petroleum-contaminated sites is depicted in Fig. 2. The dominant groups were found to be the unknown (56.45%) and the unclassified eukaryotic lineages derived from Eukaryota (34.55%), while the remaining phyla were considered insignificant (Fig. 2). These observations were similar to those of the second site where unknown sequences at the genus level derived from the phylum Eukaryota occupied the first position (37.14%), followed by unclassified sequences from phylum Eukaryota (24.07%), Ciliophora (18.17%) and Ascomycota (6.47%) (Fig. 2). A marked reduction in the quantitative number of the Tracheophyta (4.01%) and other phyla was observed in the second site (Fig. 2). Statistically, there was no significant difference at *p* < 0.05 in terms of the microbial phyla observed in these two sites.

### Taxonomic classification of microbes in petroleum oil-polluted water sites at the genus level

**Figure 3:**
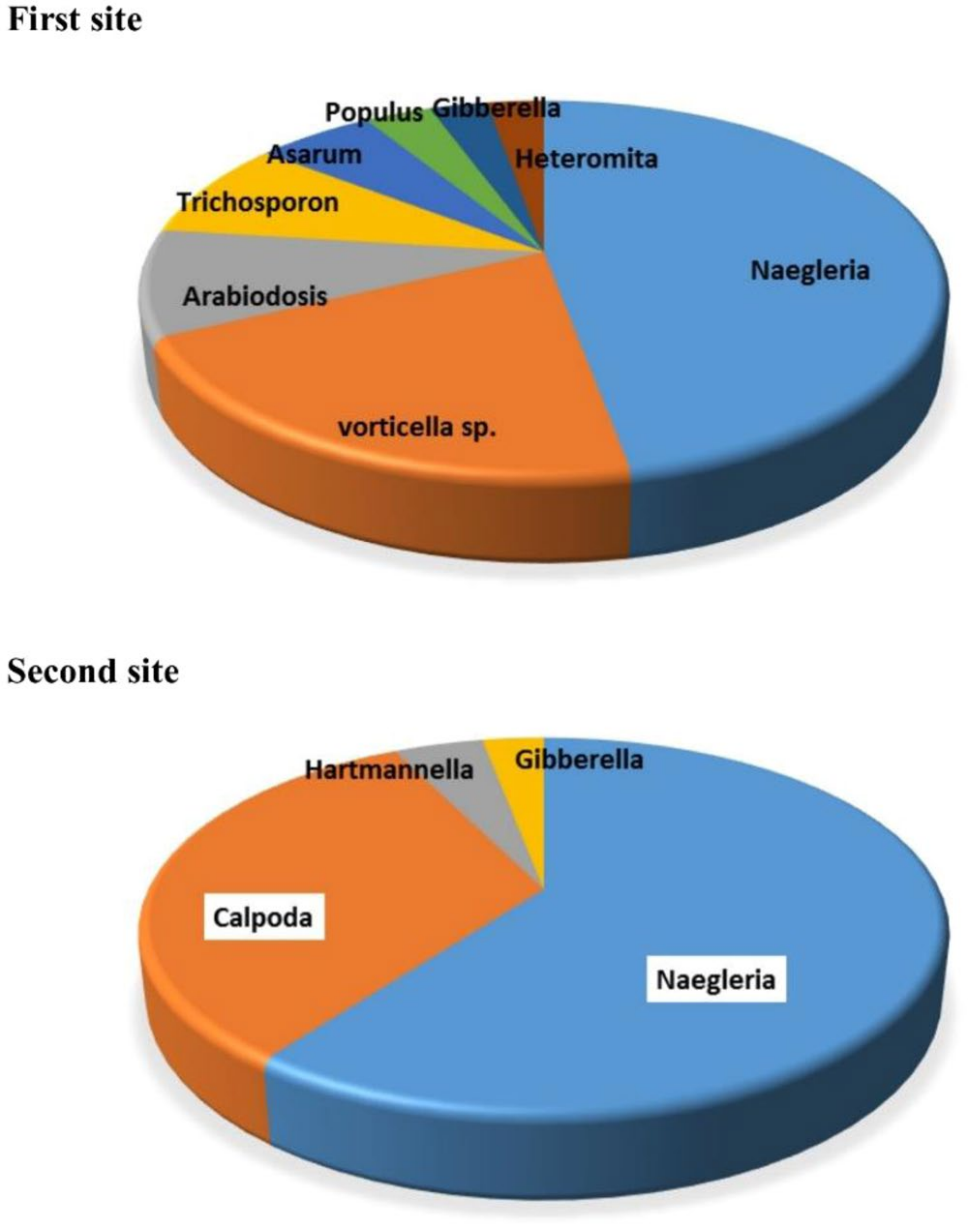
Different genera of the microbes observed in the first and second sites in petroleum-polluted water.

According to the classification at the genus level, the dominant group was *Naegleria* (15.21%) followed by *Vorticella* (6.67%) as the only ciliated protozoan genus, other species such as *Arabidopsis* (2.97%), *Asarum* (1.84%) *Populus* (1.04%) were significantly low and drastically lower in the first site (Fig. 3). Regarding the second site, the dominant group was *Naegleria* (18.29%) followed by *Colpoda* (9.86%) with the remainder of the genera representing <2% (Fig. 3). It was evident that there were few identified protozoan genus in this site due to the high number (>0%) of unclassified sequences derived from the phylum Eukaryota (Fig. 2).

## Discussion

Petroleum hydrocarbon contamination of the existing freshwater sources and the environment at large is of growing public concern as large amounts of these compounds are being released deliberately or through accidental spills^12^. Petroleum oil exploration has led to a massive release of huge amounts of oil into soil and water and the majority of these hydrocarbons are usually toxic to the end-users, as reported by Jin *et al*.^13^. In spite of these negative impacts, there are also positive aspects where the affected sites contain a diverse array of microbes, which possess unique enzymes or genes for the biodegradation of hydrocarbon contaminants^4^. According to Abbasian *et al*.^14^, the application of metagenomic analysis tools in the affected oil-spill sites provides a promising approach that allows analysis of these microbial communities and their mode of adaptation to petroleum oil contamination in the environment.

Results of the present study have demonstrated that the microbial community of the petroleum oil-polluted water aquifers of the two different sites in Pretoria/South Africa consisted of various microbial domains with the protozoa (eukaryotes) were the most dominant groups, followed by fungi and archaea which were present in low numbers in both oil-polluted aquifer sites, which were present in low numbers in both oil-polluted aquifer sites. The results obtained in this study are in line with the findings of previous studies done by Liang *et al*.^15^ and Yergeau *et al*.^16^ on metagenomic analysis of the petroleum-contaminated water obtained from the northern parts of North America and southern parts of China. According to Andrews and Floodgate^17^, protozoan microbes such as ciliates and heterotrophic dinoflagellates are mainly consumers of phytoplankton and they are also important contributors to their diet; hence, they are also able to ingest oil droplets and oil-contaminated phytoplankton. Bioaccumulation of hydrocarbon components of petroleum oil do stimulate higher uptake by protozoans and this process will therefore facilitate and enhance removal of oil from polluted water, thereby reducing the availability of oil^18^. Nevertheless, the potential role of protozoans in the interactions between dispersed petroleum oil and other aquatic microbes (biomagnification or mitigation) has generally been neglected in petroleum toxicological and bioaccumulation studies in petroleum-contaminated sites as further reported by Calbet^18^.

The representation of different phyla of microbes obtained in two petroleum-contaminated aquifer sites showed the dominant phyla to be the Eukaryota [(where a high number (>20%) of unclassified sequences were observed)] (Fig. 2). According to Atlas^19^ and Widdel *et al*.^20^, the majority of Eukaryotic family are reported to be more closely monitored in order to evaluate the potential for both aerobic and anaerobic oil biodegradation since they are known for their utilisation of petroleum hydrocarbons. Most eukaryotic organisms which are distant cousins of the protozoans such as the Ascomycota are mainly ciliates and flagellates. Kiørboe *et al*.^21^ pointed out that ciliates are common grazers in many aquatic environs and have been reported in many sites involving oil degradation or hydrocarbon-contaminated aqueous habitats. Parker^22^, Batelle^23^ and Andrews and Floodgate^17^ have reported that the protozoan phyla are well adapted to graze upon oil-degrading communities and to survive in severely polluted habitats. They reproduce rapidly and show a wide tolerance against environmental extremes in temperature, oxygen supply, pH and the presence of toxins. The phylum Ascomycota plays an important role in removing hazardous hydrocarbon compounds from water and petroleum oil from oil spills. This is the desired ecological niche for fungi, which inhabit substrates, and in biodegradation processes they are known as consumers of the carbon sources from hydrocarbons found in existing freshwater sources^10^. According to Ojo^24^, fungi have been found to be better degraders of petroleum than traditional bioremediation techniques including bacteria, and although hydrocarbon degraders may be expected to be readily isolated from a petroleum oil-associated environment, the same degree of expectation may be anticipated for microorganisms isolated from a totally unrelated environment. This phylum is also found to be capable of biodegrading a structurally diverse range of oil-derived compounds by employing a variety of mechanisms including intracellular enzymes such as cytochrome P450 monooxygenases and transferases as well as extracellular enzymes such as laccases and fungal peroxidases as reported by Harms *et al*.^25^. *Vorticella* (6.67%) was the only ciliated protozoan genus observed during this study period especially in the first site while none of the protozoa were found in the second site. This could be as a result of the high number (>30%) of unclassified sequences derived from the phylum Eukaryota in the second site which were not been able to be fully identified (Fig. 2).

## Conclusion

Based on the metagenomic data analysis, the two petroleum-contaminated sites were inhabited by a diverse array of eukaryotic (protozoa) organisms were the predominant groups, followed by fungi and other domain such as plantae which were found to be present in relatively low numbers in both sites. These microorganisms play an important role in the bioremediation process as they are found to possess rich sources of different and diverse enzymes useful for the degradation of different hydrocarbon groups; in addition, most of these microorganisms secrete surfactants that facilitate emulsification of the petroleum oil, thus enhancing the uptake of hydrocarbons into the microbial cells. The novelty of this study is that the majority of these microbes were found to be protozoa (eukaryotes) found in two sites. It is envisaged that protozoans will form part of the bioremediation process. This study thus confirms the presence of protozoan phyla, which were never before known to be playing any role in hydrocarbon degradation or inhabiting these petroleum oil-contaminated areas. The phylogenetic novelty observed during this study makes an important contribution towards a better understanding of the microbial diversity that exists in an oil-spill affected site. The findings of this study provide a scientific background and technology platform for further research and utilisation of microbial species, especially protozoan species, in petroleum-contaminated areas in South African water sources and beyond. This study also allows capacity building for water-scarce countries in developing a high level of scientific research in the area of metagenomics.

## References

[1] Haritash AK, Kaushik CP (2009). Biodegradation aspects of polycyclic aromatic hydrocarbons (PAHs): a review. J. Haz. Mater.

[2] Van Hamme JD, Singh A, Ward OP (2003). Recent advances in petroleum microbiology. J. Micro. Mol. Bio. Rev..

[3] Juhasz AL, Naidu R (2000). Bioremediation of high molecular weight polycyclic aromatic hydrocarbons: a review of the microbial degradation of benzo[a]pyrene. J. Inter. Biodeter. Biodegr..

[4] Abbasian F, Lockington R, Mallavarapu M, Naidu R (2015). The integration of sequencing and bioinformatics in metagenomics. Rev. Env. Sci. Biotechnol..

[5] Cerniglia, C. E. & Sutherland, G. R. Degradation of polycyclic aromatic hydrocarbons by fungi. In: Timmis, K. N. (Ed.), *Handbook of Hydrocarbon and Lipid Microbiology*, Springer, Berlin; London. 2079–2110 (2010).

[6] Magot M, Ollivier B, Patel BKC (2000). Microbiology of petroleum reservoirs. Mikr..

[7] Borzenkov IA, Milekhina ET, Gotoeva MT, Rozanova EP, Beliaev SS (2006). The properties of hydrocarbon-oxidizing bacteria isolated from the oilfields of Tatarstan, Western Siberia, and Vietnam. Mikr..

[8] Da Cruz GF (2011). Could petroleum biodegradation be a joint achievement of aerobic and anaerobic microorganisms in deep sea reservoirs?. AMB Exp..

[9] Uhlik O, Strejcek M, Hroudova M, Demnerova K, Macek T (2013). Identification and characterization of bacteria with bioremediation potential: From cultivation to metagenomics. Chem Listy..

[10] Thomas T, Gilbert J, Meyer F (2012). Metagenomics – a guide from sampling to data analysis. Micro. Informat. Experi..

[11] Gong JS (2013). Metagenomic technology and genome mining: emerging areas for exploring novel nitrilases. Appl. Microbiol. Biotechnol..

[12] Abbasian F, Lockington R, Mallavarapu M, Naidu RA (2015). Comprehensive review of aliphatic hydrocarbon biodegradation by bacteria. J. Appl. Biochem. Biotechnol..

[13] Jin HM, Kim JM, Lee HJ, Madsen EL, Jeon CO (2012). *Alteromonas* as a key agent of polycyclic aromatic hydrocarbon biodegradation in crude oil-contaminated coastal sediment. J. Env. Sci. Technol..

[14] Abbasian F (2016). Microbial diversity and hydrocarbon degrading gene capacity of a crude oil fields soil as determined by metagenomics analysis. J. Biotechnol. Prog..

[15] Liang Y (2011). Functional gene diversity of soil microbial communities from five oil-contaminated fields in China. J. Int. Society Microb. Ecol..

[16] Yergeau E, Sanschagrin S, Beaumier D, Greer CW (2012). Metagenomic analysis of the bioremediation of diesel-contaminated Canadian high arctic soils. PLoS One.

[17] Andrews AR (1974). & Floodgate, G. D. Some observations on the interactions of marine protozoa and crude oil residues. Mar. Bio..

[18] Calbet A (2008). The trophic roles of microzooplankton in marine systems. Ices J. Mar. Sci..

[19] Atlas RM (1981). Microbial-degradation of petroleum-hydrocarbons – an environmental perspective. Microb. Rev..

[20] Widdel, F. & Grundmann, O. Biochemistry of the anaerobic degradation of non-methane alkanes. In: Timmis, Kenneth N., McGenity, Terry J., Vander Meer, Jan Roelof, De Lorenzo, Victor(Eds), Handbook of Hydrocarbon and Lipid Microbiology, Springer Verlag, Berlin, 909–924 (2010).

[21] Kiørboe T, Tang K, Grossart HP, Ploug H (2003). Dynamics of microbial communities on marine snow aggregates: colonization, growth, detachment, and grazing mortality of attached bacteria. J. Appl. Env. Microb.

[22] Parker JG (1992). Ciliated protozoa in marine pollution studies. Ecotox. Env. Safety..

[23] Batelle, C. D. Mushrooms: Higher macro-fungi to clean up the environment. *Env. Issues**Fall*. 2000 (2000).

[24] Ojo OA (2005). Petroleum-hydrocarbon utilization by native bacterial population from a wastewater canal Southwest Nigeria. Afr. J. Biotechnol..

[25] Harms H, Schlosser D, Wick LY (2011). Untapped potential: exploiting fungi in bioremediation of hazardous chemicals. Nat. Rev. Microb..

